# Biomechanical characterization of tissue types in murine dissecting aneurysms based on histology and 4D ultrasound-derived strain

**DOI:** 10.1007/s10237-023-01759-6

**Published:** 2023-09-14

**Authors:** Achim Hegner, Hannah L. Cebull, Antonio J. Gámez, Christopher Blase, Craig J. Goergen, Andreas Wittek

**Affiliations:** 1 Personalized Biomedical Engineering Lab, Frankfurt University of Applied Sciences, Frankfurt am Main, Germany; 2 Department of Mechanical Engineering and Industrial Design, School of Engineering, University of Cadiz, Cadiz, Spain; 3 Weldon School of Biomedical Engineering, Purdue University, West Lafayette, USA; 4 Department of Radiology and Imaging Sciences, Emory University, Atlanta, USA; 5 Cell and Vascular Mechanics, Goethe University, Frankfurt am Main, Germany

**Keywords:** In vivo, Local strain, Dissecting aneurysm, 4D ultrasound strain imaging

## Abstract

Abdominal aortic aneurysm disease is the local enlargement of the aorta, typically in the infrarenal section, causing up to 200,000 deaths/year. In vivo information to characterize the individual elastic properties of the aneurysm wall in terms of rupture risk is lacking. We used a method that combines 4D ultrasound and direct deformation estimation to compute in vivo 3D Green-Lagrange strain in murine angiotensin II-induced dissecting aortic aneurysms, a commonly used mouse model. After euthanasia, histological staining of cross-sectional sections along the aorta was performed in areas where in vivo strains had previously been measured. The histological sections were segmented into intact and fragmented elastin, thrombus with and without red blood cells, and outer vessel wall including the adventitia. Meshes were then created from the individual contours based on the histological segmentations. The isolated contours of the outer wall and lumen from both imaging modalities were registered individually using a coherent point drift algorithm. 2D finite element models were generated from the meshes, and the displacements from the registration were used as displacement boundaries of the lumen and wall contours. Based on the resulting deformed contours, the strains recorded were grouped according to segmented tissue regions. Strains were highest in areas containing intact elastin without thrombus attachment. Strains in areas with intact elastin and thrombus attachment, as well as areas with disrupted elastin, were significantly lower. Strains in thrombus regions with red blood cells were significantly higher compared to thrombus regions without. We then compared this analysis to statistical distribution indices and found that the results of each aligned, elucidating the relationship between vessel strain and structural changes. This work demonstrates the possibility of advancing in vivo assessments to a microstructural level ultimately improving patient outcomes.

**Supplementary Information:**

The online version contains supplementary material available at 10.1007/s10237-023-01759-6.

## Introduction

Despite recent advancements in endovascular treatment and repair, aortic dissection and rupture remain a major clinical problem leading to between 150,000 and 200,000 deaths each year, with 10,000 occurring in the U.S. Aortic dissection develops in a weakened area of the vessel wall, often occurring in the outer one-third of the media (Benjamin et al. 2019), creating an intima-media flap separating the true and false lumens. The role thrombus plays within the false lumen, a common finding in regions with slow or stagnant flow, which is not entirely clear as it may provide protective effects on the weakened wall (Benjamin et al. 2019) or serve as a region for inflammatory cells to congregate and express proteases (Benjamin et al. 2019), possibly increasing aortic growth rates (Ruiz-Muñoz et al. 2022). Current treatment for aortic aneurysms and dissections remains insufficient by relying only on diameters and growth rates to inform treatment decisions (Glimåker et al. 1991; Kurvers et al. 2004; Satriano et al. 2015). However, there are several ongoing studies aimed at improving patient risk stratification for expansion and rupture (He et al. 2021). Investigating the relationship between the vessel biomechanics and composition helps provide a comprehensive understanding of the disease pathology.

While consideration should be taken when discerning clinical insights from experimental animal models, mice have been shown to be a useful species in which to study aortic dissection disease progression and thoroughly investigate composition changes (Busch et al. 2021). Thus, we infused angiotensin-II (AngII) into male, apolipoprotein E-deficient ($apoE^{\mathrm{\,-/-}}$) mice, an established and commonly used dissecting aortic aneurysm model (Daugherty et al. 2000). This model frequently leads to medial disruption within the first week of AngII pump implantation, creating a dissection and leftward expansion of the aorta in the suprarenal region (Goergen et al. 2011). Substantial remodeling of the vessel begins with inflammatory cell infiltration, collagen deposition, and neovascularization within the first several weeks (Daugherty et al. 2000, 2011; Busch et al. 2021).

Measuring vessel strain can characterize degenerative changes of the abdominal aortic wall such as dissections and aneurysms. The noninvasive approach presented here provides highly resolved, in vivo deformation fields of aortic tissue under physiological loading. Since wall strain relates to the elastic properties of the wall in the physiological range (O’Rourke et al. 2002; Wittek et al. 2018), the full field measurement of highly resolved local strains allows for the assessment of the heterogeneous elastic behavior of the wall and its changes during disease progression.

In the last quarter century, the most prominent and widely followed methods for estimating rupture risk and disease progression involving biomechanics are stress-based approaches (Gasser et al. 2022; Farotto et al. 2018; Vorp 2007). These can be derived from classical engineering strength approaches in which actual stresses are related to allowable stresses (Vande Geest et al. 2006). A prerequisite for a high validity of stress-based approaches is the knowledge about the in vivo unknown inhomogeneous wall thickness, geometry, and the individual material behavior. Joldes et al. (2016) were able to show, however, that if the deformed geometry is known, the stresses no longer depend on the material. With an assumption of constant wall thickness (due to missing information), the stresses in this case are then only dependent on the main radii of curvature. However, the added value compared to the currently used maximum diameter criterion is only small, since the condition of the vessel wall is completely neglected. When measuring local deformations, the state of the tissue is also unknown, but the local deformations themselves depend strongly on the state or degeneration of the wall. Measured strains thus contain much more information than just local geometry variations and can be a suitable tool to make predictions about disease progression, which is why they have been increasingly used in recent years: In vivo studies applying 4D ultrasound (Karatolios et al. 2013; Wittek et al. 2013) and cine-MRI in human subjects (Satriano et al. 2015) found heterogeneous wall strain distributions in healthy and aneurysmal abdominal aortae. Aggarwal et al. (2022), Wittek et al. (2016) and Vogt et al. (2016) also applied strains derived from 4D ultrasound to the aortic root, and Farzaneh et al. (2019) found heterogeneous distributions of stiffness properties calculated from local strain distributions of the ascending and descending aorta using gated computed tomography (CT). Multiple times it was proposed to use these distributions as mechanical biomarkers for the pathophysiological state of the wall (Wittek et al. 2018; Farzaneh et al. 2019; Aggarwal et al. 2022). Further studies demonstrated that the elastic behavior of young, aged, and aneurysmal human aortae (Derwich et al. 2016) as well as the aneurysmal sac region and neighboring non aneurysmal aortic sections (Derwich et al. 2020) can be significantly distinguished. *In vitro* and in vivo studies by Wilson et al. (2003), Romo et al. (2014) and Wilson et al. (2019) have indicated strain and distensibility as potential rupture risk predictors. The ability to investigate strain on a local level may further improve risk stratification. While direct comparison between in vivo 3D strain and wall microstructure based on ex vivo tissue analysis may not be possible in humans, animal studies such as this have the potential to provide clinical insight into patient management.

In our previous study (Cebull et al. 2019), we used 4D ultrasound to estimate 3D Green-Lagrange strain of murine dissecting abdominal aortic aneurysms. Our histological analysis revealed that areas where substantial thrombus formed and elastin fragmented were correlated with regions of low strain. Our initial observations regarding strain and composition led us to further explore and quantify this relationship. The quantitative comparison of in vivo strain imaging and histological analysis of the microstructural tissue composition in an animal model provides an opportunity to examine the extent to which the observation of heterogeneous local strain is informative about tissue heterogeneity itself. Because matrix remodeling is critical to the formation and growth of these lesions, quantification of regional material properties has the potential to increase our understanding of pathological mechanical mechanisms underlying aortic dissection.

In this study, we present an in-depth analysis of in vivo gated volumetric ultrasound data, histological analysis, and strain estimation of murine dissecting aortic aneurysms. The comparison of 4D ultrasound-estimated strain and histological analysis of vessel composition provides us with insights into how intramural thrombus deposition, elastin degradation, and collagen turnover influence in vivo kinematics. While each individual technique is useful for characterizing pathology on their own, it is the combination of noninvasive imaging with computational biomechanical analysis that is particularly useful when investigating the complex vascular remodeling processes associated with aortic dissection.

## Methods

### Animal study

We acquired imaging and blood pressures on $apoE^{\mathrm{\,-/-}}$ male mice (25.5$-$32.2 g; $12.5\pm 3$ weeks old; $n=10$) throughout a 28-day study period and excised the aortae for histological analysis (Cebull et al. 2019). At the start of the study, mice were implanted with a mini-osmotic pump (ALZET Model 2004; DURECT Corporation, Cupertino, CA, USA) that delivered AngII at a steady rate of 1000 ng/kg/min. At the end of the study, eight mice (M1-M8) were euthanized via carbon dioxide overdose. Two mice were excluded as they died due to aortic rupture prior to the study endpoint. Animals were grouped in an AAA (M1-M5) or non-AAA (M6-M8) group (Cebull et al. 2019), according to aortic diameter. All procedures were approved by the Purdue University IACUC (protocol 2002002016).

**Fig. 1:**
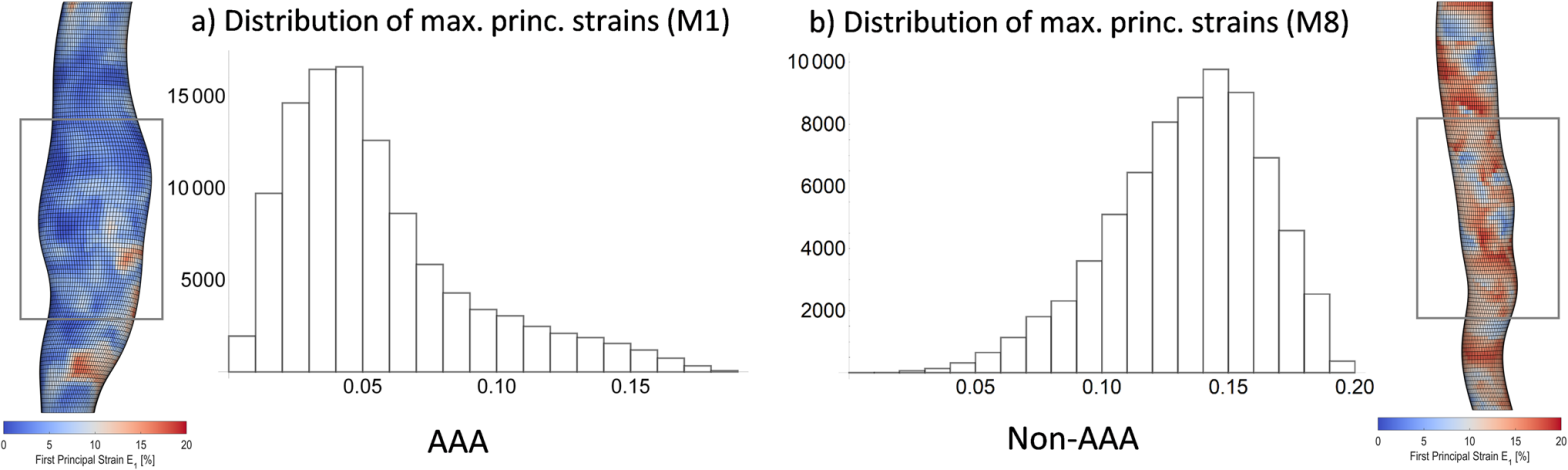
Example distributions of local 1st or maximum principal strains from aneurysmal and non-aneurysmal mouse aortae. **a** The aneurysmal aorta (left) shows a right-skewed strain distribution with a long tail of few large strains and a majority of small strain values (positive skewness = 1.191). **b** Conversely, the non-aneurysmal aorta displayed a left-skewed distribution with a long tail toward small strains and a majority of large strain values (negative skewness = $-$0.529). In aneurysmal aortae, only the strains of the aneurysmal section highlighted within the box were included in the evaluation. For non-aneurysmal aortae, we selected a corresponding section of suprarenal abdominal aorta

**Fig. 2:**
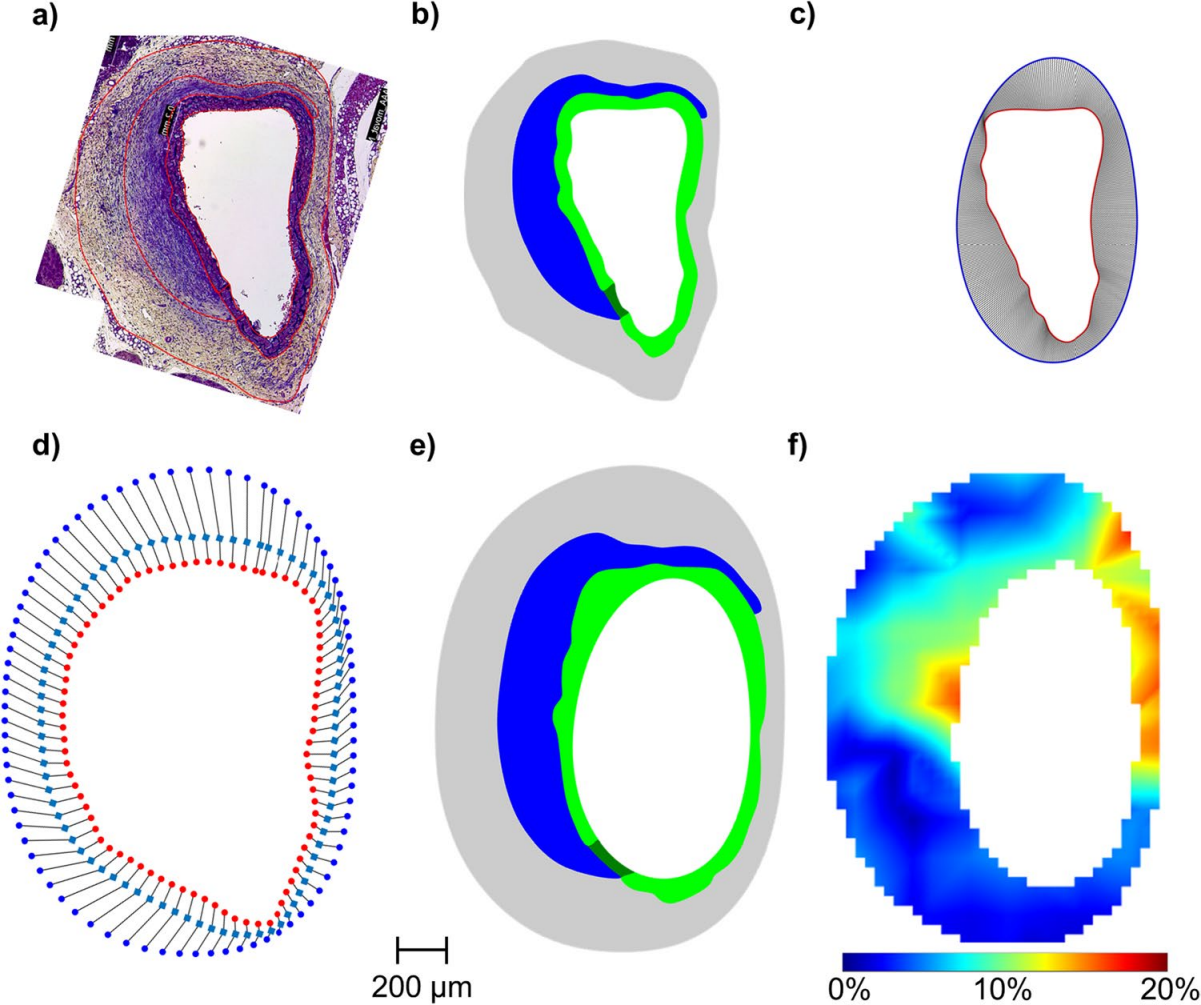
Overview of the workflow for the registration of histological and strain imaging data. **a** Manual segmentation of the distinct regions from the histological images. **b** The meshes created from the histology segmentation in an undeformed reference configuration. **c** Registration of lumen geometry from histology (red, inner contour) with in vivo lumen geometry (blue, outer contour). **d** Registration of the outer geometry from histology (red dots, inner contour) with the in vivo outer geometry (blue dots, outer contour). Here, registration was done in two steps. The manually drawn middle contour (light blue, squares) was used as an auxiliary contour for a first registration. The results of this first registration from the histology contour (red dots) to the auxiliary contour was then used as a starting point for a second registration step to the actual outer geometry (blue dots). The point density in (**d**) is reduced for clarity. In (**c**), the point density is not reduced. **e** Geometry after applying displacement boundary conditions from registration of lumen (**c**) and outer contour (**d**) using FEA from the deformed configuration. **f** Superposition of the deformed contours of all regions found in the FE analysis with the in vivo strains from ultrasound imaging. Scale bar refers to **a**–**f** and the colorbar refers to (**f**)

### Image acquisition, processing, and analysis

Our full data acquisition description can be found in Cebull et al. (2019). Briefly, we used a high-frequency ultrasound system (Vevo2100 Imaging System; FUJIFILM VisualSonics, Inc., Toronto, ON, Canada) to collect 4D data at the end of the study. For mice where an aneurysm was visibly present (>50% diameter growth), we acquired 4D data 14 days of post-aneurysm diagnosis. For the mice below the 50% threshold, we collected the data at 28 days of post-pump implant. We acquired the images using a 40 MHz center-frequency 256-element array transducer (MS550D, 40 µm axial resolution, 90 µm lateral resolution) via a long axis acquisition. The 4D ultrasound data were created by compiling ECG-gated kilohertz visualization (EKV) loops, collected at 1000 frames-per-second, into a time-resolved 3D dataset. We used a linearly translating step motor to acquire the EKV loops every 80 µm, capturing the entire aorta in our region of interest from below the renal arteries to above the diaphragm.

We processed the 4D ultrasound images using a custom MATLAB code (R2018a, Mathworks Inc., Natick, MA, USA), resampling the volumetric data into isotropic voxels (40 µm$^3$) (Damen et al. 2021). To estimate the 3D strain, we used the direct deformation estimation (DDE) approach described previously (Boyle et al. 2019). This approach estimates deformation by using a reference volumetric image (end diastole) and applies an optimized warping function Eq. (1), which is a 3D affine transformation using a warping parameter, $\varvec{p}$, to successive time points that minimize the voxel intensity differences throughout the cardiac cycle.

1 $$\begin{aligned} \varvec{W}(x;\,\varvec{p}) = \begin{bmatrix} 1 + p_1 &{} p_4 &{} p_7 &{} p_{10} \\ p_2 &{} 1 + p_5 &{} p_8 &{} p_{11} \\ p_3 &{} p_6 &{} 1 + p_9 &{} p_{12} \\ 0 &{} 0 &{} 0 &{} 1 \end{bmatrix} \begin{bmatrix} x \\ y \\ z \\ 1 \end{bmatrix} \end{aligned}$$

Because the warping function is directly analogous to deformation, the deformation gradient tensor (the first nine elements of the warping function, $\varvec{W}$) is known without consideration of displacement fields (Boyle et al. 2019). We used the gradient tensor to calculate maximum principal Green-Lagrange strain, which we visualize as an in vivo, 3D strain map overlaid on the image data.

### Strain distribution indices

In order to quantify the differences of the calculated maximum principal Green-Lagrange strain distributions, the following strain distribution indices (SDI) characterizing these data were determined:

- The arithmetical mean of the strain distribution,
- The maximum local strain value,
- The coefficient of variation, i.e., the ratio of the standard deviation and the mean of the strain distribution as a measure for its heterogeneity (“heterogeneity index”).

In addition to these SDI metrics that were previously proposed as mechanical biomarkers for the pathological state of the aortic wall (Karatolios et al. 2013; Derwich et al. 2016; Wittek et al. 2018), the skewness of the strain distribution was also determined in this study (cf. Fig. 1). A positive skewness value indicates a right-skewed distribution characterized by a long tail of few large local strain values. In contrast, a negative skewness value is associated with a left-skewed distribution with a long tail toward small strains and a majority of larger strain values. SDIs were computed from local strain value distributions using Wolfram Mathematica (v10.3, Wolfram Research, Champaign, IL, USA). For all SDIs, hypothesis tests were performed on the AAA and the non-AAA group with null hypothesis that the means or medians of the two datasets are equal using the function LocationTest of Wolfram Mathematica that examines both of the tested datasets for normality first and then chooses the most powerful hypothesis test that applies to the datasets. In each case, a Student’s *t*-test for univariate data was performed.

### Aortic tissues histology and staining

The aortae were preserved immediately following euthanasia of the mice using 4% paraformaldehyde (initial 72 h) and 1x phosphate-buffered saline solution stored at 4 $^\circ$C. We embedded the aorta in paraffin from the renal arteries to the diaphragm, then took cross-sectional slices (5 µm) at multiple locations throughout the embedded region. For the purpose of this study, we chose two locations in each aorta for our in vivo strain to histology comparison. We stained adjacent slices with hematoxylin and eosin (H &E) and Movat pentachrome, which identifies elastin (black), fibrin and red blood cells (bright red), collagen (yellow), GAGs (blue), and muscle/cytoplasm (purple). The tissue slices were visualized at 40x magnification on the Leica ICC50 W microscope (Leica Microsystems GmbH, Wetzlar, Germany).

### Data registration

#### Segmentation of the ultrasound images

The individual aneurysm regions are not clearly identifiable in the ultrasound images, so only the recognizable outer aneurysm wall and lumen were manually segmented. The contours of the lumen and outer wall themselves were isolated from the images in MATLAB R2022b with the edge detection function edge using the Soebel method and then interpolated using biharmonic splines. This results in the contours of the wall and lumen based on the ultrasound measurements.

#### Segmentation of the histological images

The histological images of the aneurysm wall were manually segmented into areas based on composition by the same experienced observers (cf. Fig. 2a). The areas were divided into elastin with and without thrombus attachment (green), fragmented elastin (dark green), and thrombus with (red) and without (blue) red blood cells (RBCs). Not all areas were present in all section planes. The contours of each area were exported as pixel values and imported into MATLAB. After converting the pixel values into millimeters, the number of points describing the segmented contours was increased from the previous approximately 50 points to 1,000 points using biharmonic spline interpolation. The resulting contours of the distinguished areas based on the histological stains where than imported into HyperMesh 2022.1.

#### Meshing of the segmented contours

Meshes were then built from the individual contours based on the histological segmentations (cf. Fig. 2b). For this purpose, the interpolated contours were imported into HyperMesh 2022.1 (Altair Engineering Inc., Troy, Michigan, USA), from which contiguous areas were first constructed. Each surface of the areas was individually meshed from mixed triangular (S3R) and quadrilateral (S4R) shell elements. The areas with elastin and fragmented elastin are significantly smaller compared to all other areas and were therefore more finely meshed. The mean element edge length in the elastin and fragmented elastin regions was $4.00 \pm 0.36$ µm and in all other regions $8.60 \pm 1.94$ µm (Mean ± SD, respectively). The resulting nodes of the lumen and outer wall contours were then isolated and initially loaded back into MATLAB for registration to the ultrasonic geometry contours.

#### Registration of histological and ultrasound contours

The isolated contours of the outer wall and lumen from both imaging modalities were registered individually in the next step. A coherent point drift (CPD) algorithm with selected non-rigid transformation was used, which is implemented in MATLAB as pcregistercpd function. By varying the two parameters InteractionSigma and SmoothingWeight, the histological contours were deformed to best fit the ultrasound contours. To achieve a better registration result, the ultrasound contours were previously increased to 20,000 points using biharmonic spline interpolation. The centroids of the two lumen contours were superimposed as the initial value of the registration algorithm. Contours for which registration in one step did not lead to good results due to the alternation of concave and convex segments were registered in two or three steps. For this purpose, auxiliary contours were manually drawn between the histological contours and the ultrasound contours. Starting from the histological contour, a registration to the first auxiliary contour was performed in the first step. The result of this registration was then used as the starting point for a subsequent registration, and this procedure was repeated until a good registration of the actual ultrasound contour was possible. 65% of the contours had to be registered in three steps, 25% in two steps, and 10% could be registered in one step. An example of a one-step registration is shown in Fig. 2c and for a two-step registration is shown in Fig. 2d. The registration was considered successful when the root mean squared error (RMSE) for the lumen contours was <0.2 µm and for the outer contours <0.75 µm. Additionally, it must be fulfilled that there is no change in the order of the contour points, which can happen if the contours to be registered have strong curvature,and the points of the histological contours were registered as evenly as possible onto the ultrasound contours. The mean RMSE between the registered contours was $0.09 \pm 0.07$ µm for the lumen and $0.21 \pm 0.28$ µm (mean ± SD, respectively) for the aneurysm walls. As a result, the displacements of each node of the wall and lumen contours generated in the meshes were exported as csv-files.

#### Finite element analysis to find the ultrasound contours

2D plane stress finite element models were generated from the meshes using Simulia Abaqus 6.12 (Dassault Systemés, Vélizy-Villacoublay, France). The results of the registration of the outer and lumen contours provide the displacements of these contours from the load-free configuration to the loaded in vivo configuration. The mechanical problem solved by a FEA software can thus be described as follows:

- **Known:** initial position of the entire domain (as given by the histological images) and current position of the outer and lumen boundaries (as imaged by ultrasound), i.e., the displacement field of the outer and inner boundaries of the aneurysm that maps the "histological" onto the "ultrasound" configuration of the cross section. No surface tractions are applied, load is defined through the prescribed deformation!
- **Unknown:** deformation field within the domain, in particular, current position of the boundaries of blue/red thrombus as well as elastin/fragmented elastin.

Problems of this type have been previously referred to as “displacement - zero traction” problems (Miller 2005; Wittek et al. 2007, 2009). These are special cases of “displacement – traction” problems (Ciarlet 1988). Wittek et al. (2005) and Wittek et al. (2007) applied nonlinear finite element procedures in computing brain deformation for image-guided neurosurgery and showed that accurate computation of brain deformation during craniotomy-induced brain shift can be done by defining load through the prescribed displacements, the same type of problem as present in this study. Furthermore, Wittek et al. (2009) were able to show that when using an appropriate finite deformation solution, the choice of constitutive model and its parameters do not affect the accuracy of the calculated displacements of internal structures when solving “displacement – zero traction” problems. Our cases all correspond to such “displacement - zero traction” problems; the load is defined by the displacement fields of the lumen and outer contours, which are completely known using the previous registration results. So the calculated displacements from the registration routine were applied as displacement boundary conditions to the nodes representing the (histological) lumen and wall contours. Because the choice of constitutive model and parameters have little effect on the calculated displacements, a very distensible hyperelastic Neo-Hooke strain energy function with $C_{10}=1$ GPa and $D=0$ was chosen likewise for all components of all cross sections. The performed analysis mapped the deformed lumen and wall contours that were generated based on the segmentation of the histological images onto the in vivo configuration of the contours as obtained from strain imaging. Depending on the prescribed deformation of the contours, the mesh of the cross section and segmented areas that corresponded to different tissue types was deformed. This deformed mesh provided an estimate of the in vivo configurations of the different tissue components though the computed strains and stresses were purely artificial. Correspondingly, for subsequent further evaluation only the deformed contours, described by the positions of the nodal coordinates, which resulted from the FE analysis of the individual areas, were used.

**Fig. 3:**
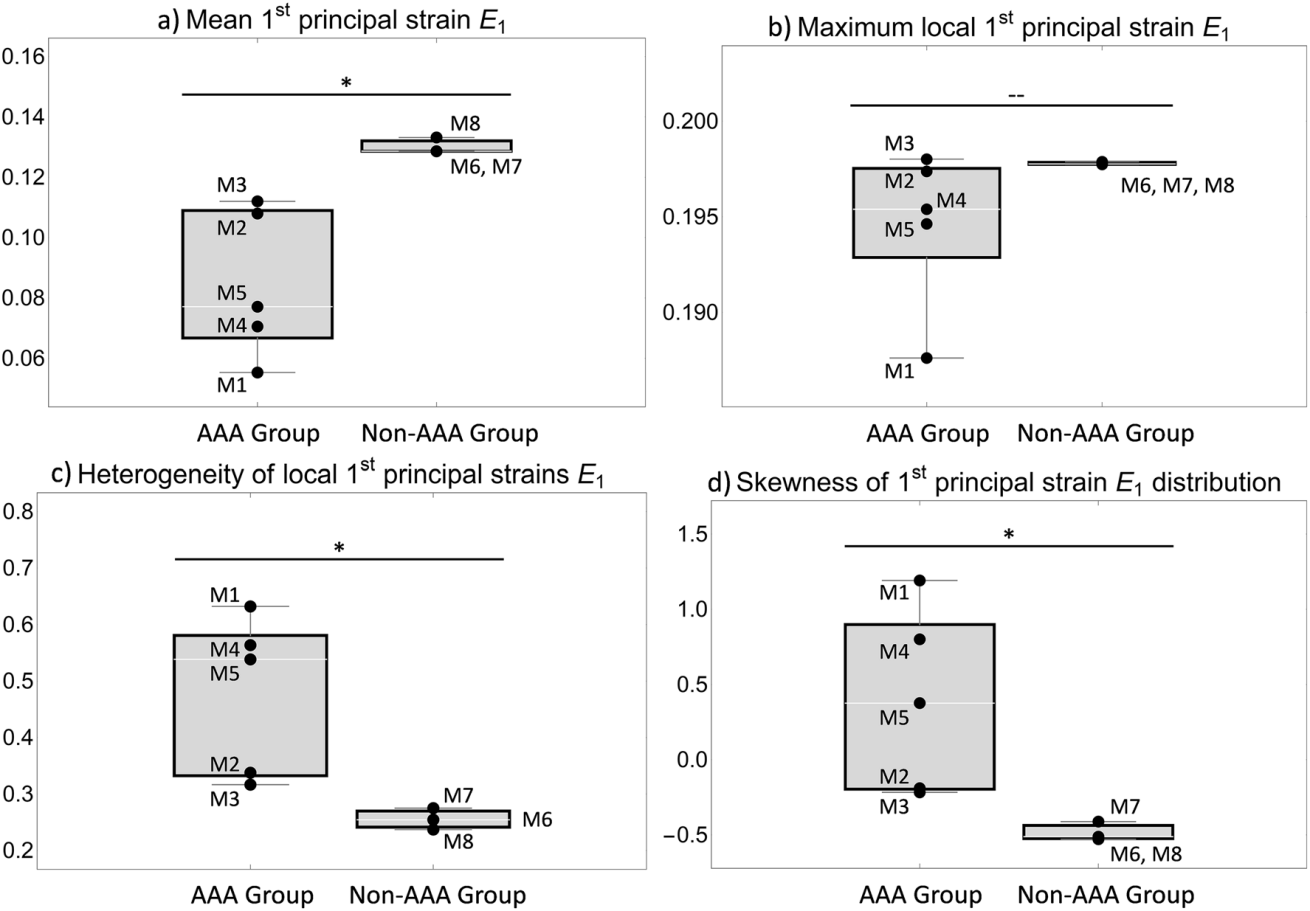
Comparison of the indices characterizing the distribution of local $1^{\textrm{st}}$ principal strain values that were obtained for aneurysmal (“AAA Group”) and non-aneurysmal (“Non-AAA Group”) mouse aortae: **a** mean, **b** maximum, **c** coefficient of variation as measure for heterogeneity and d) skewness. In all cases, a Student’s *t*-test was performed. * indicates a significant difference between both groups corresponding to $p<0.05$ and—indicates that no significant difference was observed

**Fig. 4:**
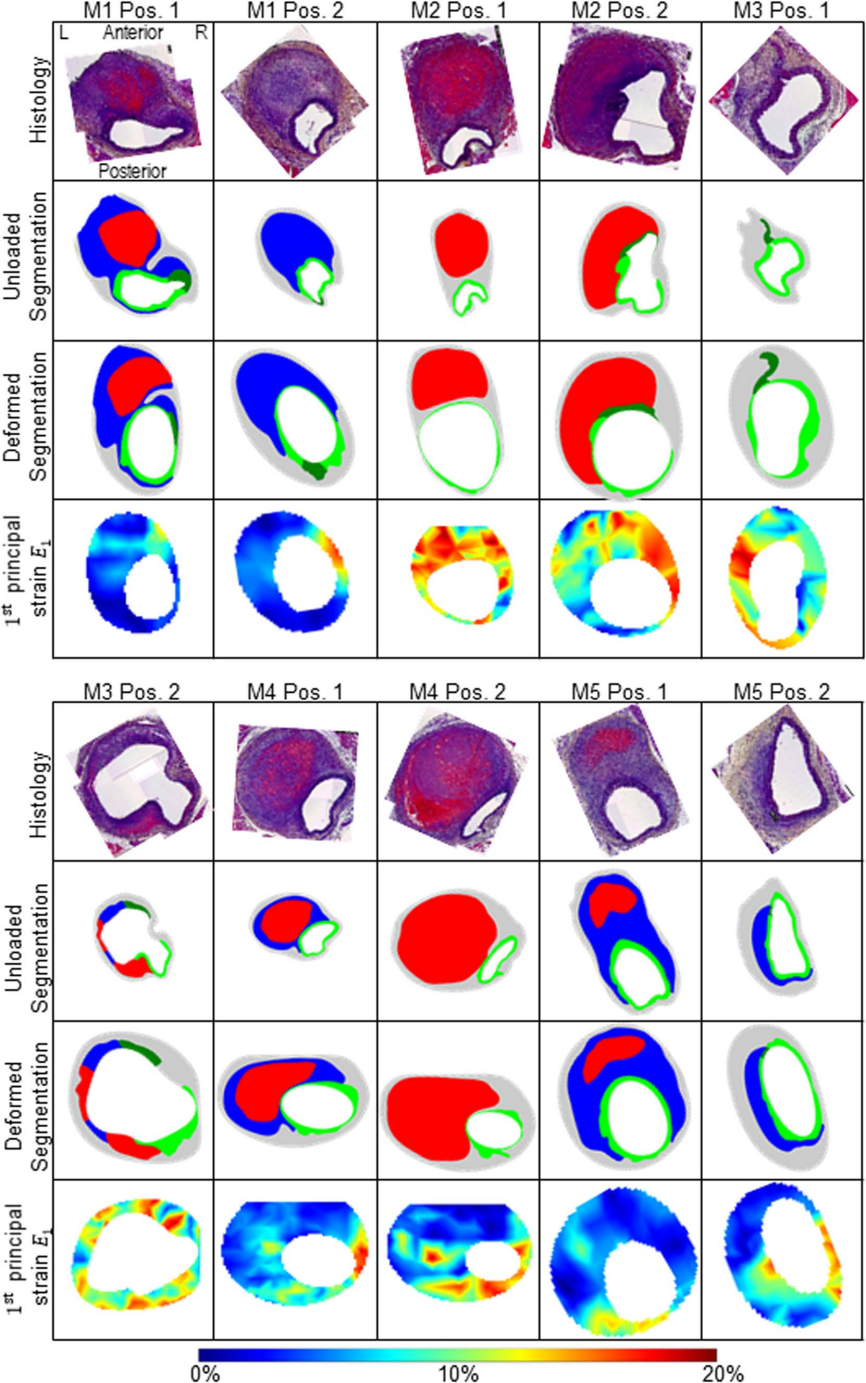
Overview of all five mice (M1-M5) with two section positions each (Pos. 1 and Pos. 2). In the first row the histological staining (Histology, Movat Pentachrome), in the second row the undeformed configuration based on the histological staining of the first row (Histo. Config.), in the third row the deformed in vivo configuration (in vivo Config.) and in the fourth row the in vivo strains, respectively. L indicates the left position and R the right position of the vessel. The position information L, R, anterior, and posterior refers to all images in this figure. The colored segmentations in the second and third row are divided into: gray: outer area (adventitia and surrounding tissue); light green: elastin intact; dark green: elastin fragmented; blue: thrombus without red blood cells; red: thrombus with red blood cells. The colors of the strain plots in the fourth row correspond to the colorbar ranging between 0 and 20% $1^{\textrm{st}}$ principal strain $E_1$. Images are not scaled equally

**Fig. 5:**
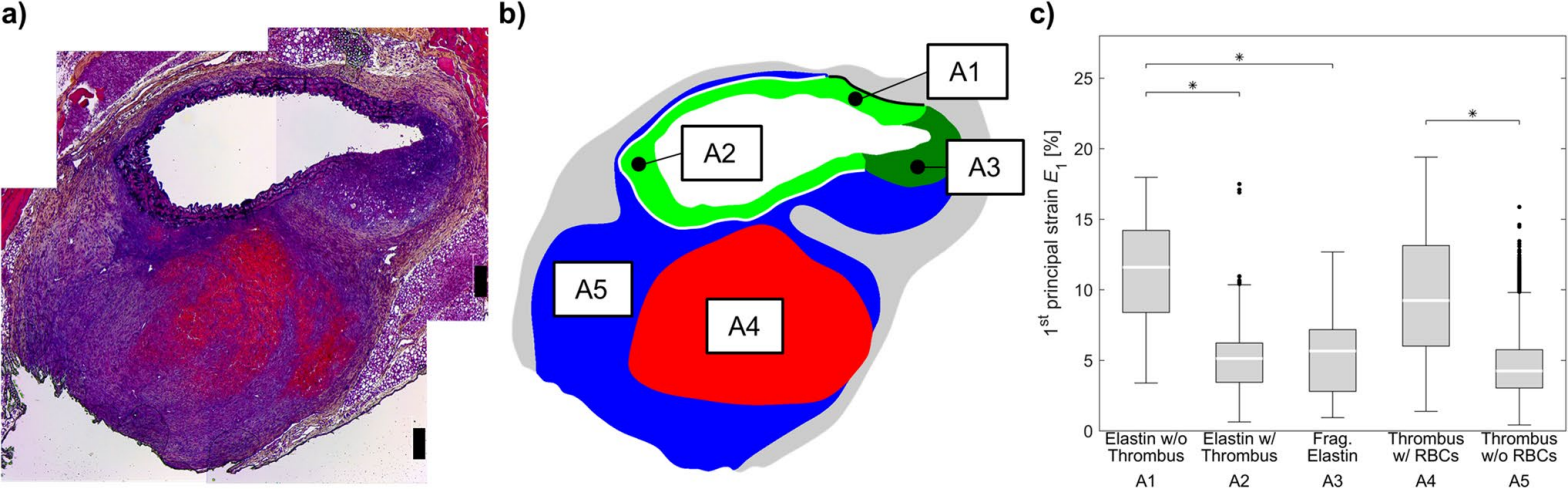
**a** Histology image and **b** segmentations of the differentiated aneurysm areas (A1-A5). **c** Box-Whisker plots of strains in the differentiated aneurysm areas (A1–A5). “Elastin w/o” and “w/ thrombus” describes the regions in elastin without and with adjacent thrombus (A1 [black border, no adjacent thrombus] and A2 [white border, with adjacent thrombus]). “Fragmented (Frag.) Elastin” describes the regions where fragmented elastin is present (A3). Similarly, “thrombus w/” and “w/o red blood cells (RBCs)” describes regions with varying levels of erythrocytes within the intramural thrombus (A4 and A5). * indicates a significant difference between groups at the 5% level with Bonferroni correction

#### Isolation of the strains of the individual aneurysm areas

In order to be able to separate the strains of the differentiated areas, the deformed contours found by FE analysis were superimposed with the ultrasound strain images (cf. Fig. 2f). The strain values, which were within the respective area contours, were then isolated, grouped, and compared to each other. Note that these are the strain values obtained by 4D ultrasound imaging and not the artificial ones resulting from the FE analyses.

### Statistical evaluation

The groups with the strains of the individual areas were then statistically compared. Elastin with and without thrombus attachment, intact elastin with fragmented elastin, thrombus with and without RBCs, and thrombus with RBCs and intact elastin were compared. A Mann–Whitney U-test was performed for this purpose. Because of the comparison of five groups, differences were considered to be statistically significant at the 5% level with Bonferroni correction.

## Results

The diastolic-systolic strain amplitude was analyzed for each animal at end of study. The mean and maximum value as well as the heterogeneity index and the skewness of the strain distribution of the first principal Green-Lagrange strain are shown in Fig. 3. The maximum local strains are similar in all animals, whereas the mean strain is significantly lower in the AAA group compared to the non-AAA group ($p<0.05$). The heterogeneity index is increased in the AAA group ($p<0.05$). The skewness of the distributions also significantly differentiates the two groups. In the non-AAA group, consistently negative skewness values <$-$0.4 were found indicating pronounced left skewed distributions. In contrast, in the AAA group, either positive skewness values were observed indicating right skewed distributions or small negative values (M2: $-$0.19, M3: $-$0.22). Within the AAA group, animals M2 and M3 showed values that were close to the non-AAA group in all parameters analyzed. The wall microstructure (*histology*), the segmented wall components in unloaded (as derived from histological sections, *unloaded segmentation*) and deformed (mapped onto the sections from in vivo imaging, *deformed segmentation*) configurations as well as the systolic strain distributions ($1^{\textrm{st}}$ *principal strain* $E_1$) are shown in Fig. 4.

For further analysis, the wall strains were grouped according to the tissue regions as described by the deformed segmentations. The resulting strain distributions are shown in Fig. 5c, strain distributions of all 10 single datasets can be found in Appendix A. Medial wall strains were analyzed for regions containing disrupted and intact elastic lamellae. The latter were further differentiated according to the presence of thrombus on the abluminal side of the media. Thrombus regions were separated as those with or without red blood cells. The comparison of the strain distributions grouped according to tissue types showed that strains were highest in areas containing intact elastin without thrombus attachment. In contrast, in areas with intact elastin and thrombus attachment ($p<0.001$) as well as areas with disrupted elastin ($p<0.001$), we found significantly smaller strains. Strains in thrombus regions with RBCs were significantly higher compared to thrombus regions without RBCs ($p<0.001$). The strains in thrombus regions with RBCs were similar to those in wall regions with intact elastic lamellae without thrombus attachment ($p=0.098$). Also, strains in thrombus regions without RBCs were similar to those in wall regions with intact elastic lamellae with thrombus attachment ($p=0.062$) and fragmented elastin ($p=0.091$).

**Fig. 6:**
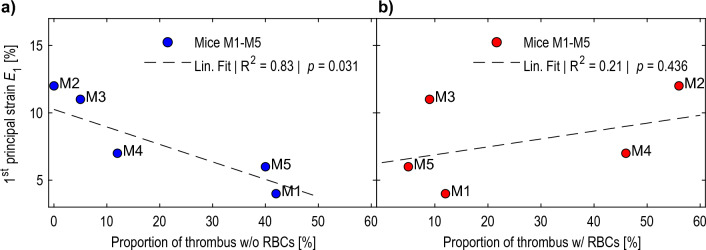
Mean $1^{\textrm{st}}$ principal aneurysm strains $E_1$ of mice M1-M5 over the proportion of **a** thrombus without red blood cells (RBCs) and **b** thrombus with RBCs relative to total wall cross section averaged from the cross-sectional images. The dashed lines show the linear fit with an $R^2=0.83$ for thrombus without RBCs ($p=0.031$) and an $R^2=0.21$ for thrombus with RBCs ($p=0.436$)

We found that strains averaged from the cross-sectional images show a strong linear correlation ($R^2=0.83$, $p=0.031$) with the proportion of thrombus without RBCs, indicating a reduction in mean strain with greater proportion of thrombus without RBCs (cf. Fig. 6a). For thrombus areas with RBCs no strong linear relationship could be found ($R^2=0.21$, $p=0.436$, cf. Fig. 6b).

## Discussion

Vascular diseases, including atherosclerosis, aortic aneurysms, and aortic dissections, are typically associated with increased heterogeneity of local biomechanical properties of the wall. Medial degeneration, remodeling of a neo-adventitia, invasion of lipids, and inflammatory cell migration into the wall (Niestrawska et al. 2019) contribute to this heterogeneity as well as “new” tissue components such as calcified plaques and intraluminal thrombus of different age and composition. The ability to assess these differences and monitor the changes in wall composition, namely the elastic behavior and their increasing heterogeneity in a noninvasive, in vivo approach might provide valuable information on individual disease progress and rupture risk. Eventually, this could help contribute to better informed clinical decision making on elective aneurysm elimination by open surgery or endovascular treatment. Thus in recent years several groups have proposed the use of in vivo full field strain imaging for gathering additional patient-specific information on degenerative changes and disease progress beyond the maximum diameter criterion (Bersi et al. 2016; Cebull et al. 2019; Derwich et al. 2016, 2020; Forneris et al. 2020, 2021; Karatolios et al. 2013; Satriano et al. 2015; Wittek et al. 2018).

Based on experimental data of a previous animal study (Cebull et al. 2019), the objective of this current study was to examine the potential of in vivo strain imaging for determining the differences in the structural composition of the murine aortic wall after formation of a dissecting aortic aneurysm. To do so, we have followed two approaches. First, we quantified the relations between different tissue components and in vivo principal strain values measured in the corresponding regions of the dissecting aneurysms. Second, we tested whether previously proposed statistical indices from the obtained SDIs are capable of detecting differences between aneurysmal and non-aneurysmal aortae, i.e., aortae exhibiting different microstructural composition assessed via histology.

Our results show that in vivo principal strain analysis can be used to distinguish tissue regions with different microstructural composition: intact elastin could be separated from fragmented elastin and thrombus without RBCs (cf. Fig. 5c A1, A2, A3, A5). Wall regions that were composed of intact elastin and where the deformation was not limited by attachment to stiffer thrombus regions displayed significantly larger strains compared to those with thrombus attached (cf. Fig. 5c A1, A2). In regions of thrombus with high content of fibrin and RBCs that appear red in the histological staining there were significantly larger deformations under physiological loading compared to “blue” thrombus where no RBCs or fibrin could be identified via histology. However, the relationship between strain values and tissue type is not unique: Intact elastin without thrombus attachment and thrombus with RBCs showed similar strains. The same was true for intact elastin regions with attached thrombus, fragmented elastin, and thrombus without RBCs. Thus this approach provides useful insight, but does not give a unique pattern to each distinct region of dissecting lesions.

We assumed that the similarities in the measured strain distributions reliably reflect similarities in the elastic behavior. Examining the dependency of the elastic properties of human *intraluminal* thrombus (ILT) on age, Tong et al. (2011) distinguished four phases of thrombus evolution: very fresh, young, intermediate, and old. Performing biaxial extension tests on the luminal, medial, and abluminal layers of ILT, they were able to show that stiffness of the luminal layer increases significantly from young through intermediate to old ILTs. Microstructurally, young thrombus is characterized by a high content of erythrocytes that are caught in a loose network of thin fibrin bundles (Spiewak et al. 2022). This composition is in line with what can be identified from histological images for the *intramural* thrombus with RBCs (red regions, IMT) found in aortic dissections in the current study. According to Tong et al. (2011), the stiffer “intermediate” and “old” ILT tissues are characterized by disruption of erythrocytes, a more condensed fibrin network with thick bundles, and the eventual disruption of the fibrin network and condensation of the residue proteins. This fits well with the characteristics of thrombus without RBCs (blue regions) in the current study as no erythrocytes could be identified in this portion of the IMT from the histological sections. Moreover, no red fibrin could be detected in the older portion of the IMT.

**Table 1:** Results of error estimation of in vivo contours. Similarity Index (SI) gives the similarity of the resulting areas based on the simulations with constant and varied material parameter $\mu$. The difference of the resulting median strains based on these two areas is given by Strain Diff. and the relative error of the median strains is given by Rel. Error. T. w/o RBCs refers to regions containing blue thrombus (w/o red blood cells) and T. w/ RBCs refers to regions containing red thrombus (w/ red blood cells). All values are given as mean ± standard deviation

Region	SI [%]	Strain Diff. [%]	Rel. Error [%]
Elastin	$92.9\pm 2.9$	$0.041\pm 0.204$	$1.78\pm 4.22$
Frag. Elas	$99.2\pm 0.2$	$0.008\pm 0.135$	$2.10\pm 5.64$
T. w/o RBCs	$91.2\pm 3.8$	$0.228\pm 0.513$	$3.95\pm 7.72$
T. w/ RBCs	$90.9\pm 0.8$	$0.010\pm 0.107$	$0.71\pm 2.07$

Our results may be impacted by limitations of the developed method for the alignment of strains and histological data. The grouping of strain values for the distinguished regions A1-A5 (cf. Fig. 5) was done based on the segmentation of these regions in histological images. In histology, however, only a thin slice of the aortic cross section was examined. In addition to the fact that this slice necessarily was not pressurized, its configuration may be affected by the preparation of the sample. In contrast, ultrasound strain imaging showed the pressurized in vivo configuration of the corresponding cross section. This necessitated the registration of the tissue areas that were segmented from histology onto the in vivo configuration that was obtained from ultrasound. Then, ultrasound strain values were grouped using the deformed contours of these very regions. Only the inner lumen contours and the outer wall contours were given by displacement boundary conditions found from the registration. The contours of the distinguished regions were then obtained from the resulting deformation of the meshes using FEA.

**Fig. 8:**
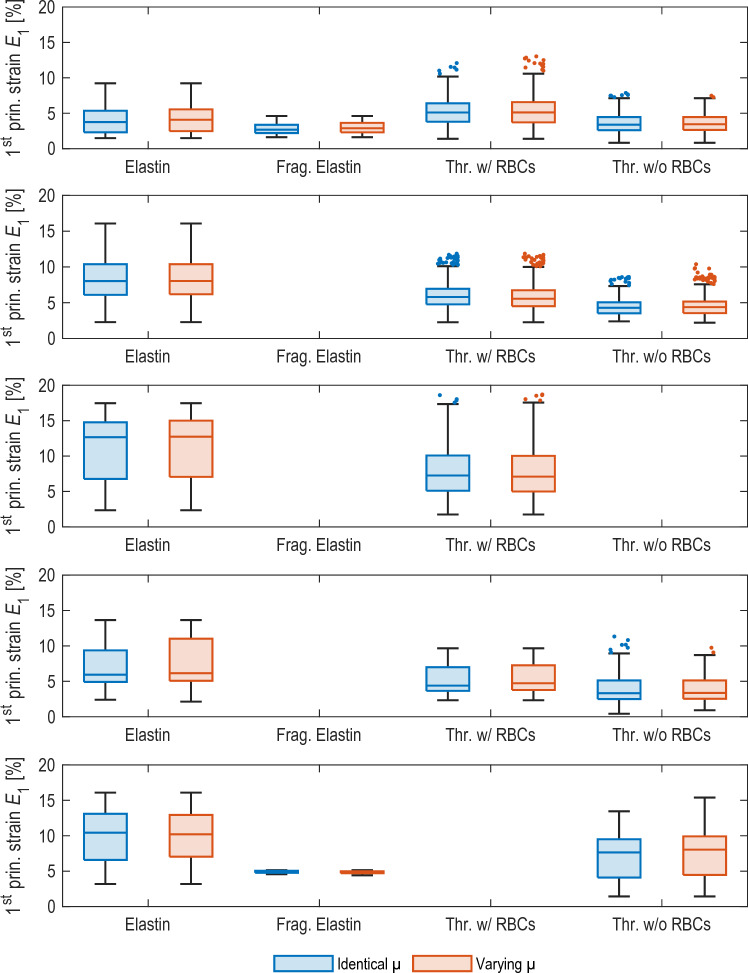
Results of the obtained strains for the error estimation of the inner contours. For one of the slices of each of the five mice M1-M5 (row 1 to 5), the simulations were performed with constant material parameter $\mu$ (Identical $\mu$, blue, left box, respectively) and corresponding tangent stiffnesses for the different tissue regions (Varying $\mu$, red, right box, respectively). Based on the resulting contours, strains were then isolated and compared using box-whisker plots. The quantified error estimates can be found in Table 1

Because the material behavior must be known to use more common pressure-based simulations, we decided against them. However, as described in Sect. 2.5.5, our data meet the requirements for “displacement—zero traction” simulations. Functionality of these simulations was demonstrated in Miller (2005), Wittek et al. (2005, 2007) and Wittek et al. (2009). Accordingly, the resulting contours for the individual tissue regions in this type of simulation are only weakly dependent on the material equations or material parameters used (Wittek et al. 2009). We therefore used the hyperelastic Neo-Hooke material equation with identical stiffnesses ($\mu = 1$ GPa) to find the in vivo contours of the individual regions. To estimate the error of the contours (and the strains isolated based on these contours), we repeated the simulations using characteristically different material properties for each tissue type: To the individual regions, we assigned estimated stiffnesses based on tangent moduli from tensile tests on these tissue types. Thrombus w/ RBCs showed the lowest stiffness by a tangent modulus of approximately 65 kPa (O’Leary et al. 2014). Thrombus w/o RBCs was modeled as 2.5x stiffer (O’Leary et al. 2014), elastin as 10x stiffer (Nightingale et al. 2022), and fragmented elastin as 15x stiffer (Nightingale et al. 2022). Based on these contours, strains were then also isolated and compared to the isolated strains based on the contours with identical stiffnesses. The resulting areas of both simulations were compared using the Dice coefficient or similarity index (SI), which is a measure for the percentile overlap between both areas. It is calculated by

2 $$\begin{aligned} \text {SI} = 2\cdot \frac{P_C \cap P_V}{P_C + P_V}\cdot 100\% \quad , \end{aligned}$$

with $P_C$ and $P_V$ being the number of pixels of the areas described by the two contours, respectively. The SIs for the resulting areas between $90.9-99.2\%$ show very good agreement of the calculated contours. The differences of the median strains isolated based on the two contours show values between $0.010-0.228\%$ as well as relative errors between $0.71-3.95\%$. These results show that it is appropriate to apply “displacement - zero traction” simulations in the current study and confirm the findings by Wittek et al. (2009) that the results of this type of finite element analysis depend only weakly on the chosen constitutive behavior. The quantified differences of the different regions can be found in Table 1, box-whisker plots of the isolated strains for both simulations can be found in Fig. 8. The found significances between the different tissue regions and significance levels (see Ch. 3 and Fig. 5c) are identical for both simulations.

While the lumen position is readily apparent in both ultrasound imaging and histological sections, the greatest uncertainty lies in the determination of the outer adventitial vessel wall contours from both techniques. In the methodology used here, however, the outer vessel wall contour has a direct influence on the resulting deformed contours of the distinguished regions. The strains in the directly adjacent areas of the vessel wall (cf. Fig. 4, gray areas) were therefore neglected. The influence of the registration, in turn, is negligible compared to the manual determination of the vessel wall contour, since all histological contours could be registered uniformly and with minimal mean deviations to the ultrasound contours (root-mean-squared error smaller than 0.1 µm or 0.21 µm for a lumen diameter of approx. 1,000 µm, see Ch. 2.5.4). From the error calculation, it can be estimated that a difference in the resulting areas of 1% due to uncertainties in the segmentation results in a difference in the median strains of 0.01% or a relative error of 0.33%.

The area edges resulting from the deformation of the meshes, however, do not appear physiological in all cases. Especially for the areas of intact and fragmented elastin of M1 pos. 1 and 2, M3 pos. 2, and M4 pos. 2 where one would expect more uniform, smoother area edges with fewer bumps (cf. Fig. 4). But due to the lower ultrasound resolution (and resulting strains) compared to the histological sections, the influence of the area edges should have little effect on the subsequent grouping of strain values for the distinguished regions.

SDIs were used to condense the information inherent in the distributions of heterogeneous local strain values and to characterize these metrics beyond a mean value. Apart from the local maximum strain, all of these indices significantly differentiated the expanded AAA group from the non-AAA group, without overlapping specimens. An additional distinction was observed within the AAA-group between mice M2 and M3 as their SDIs were found to be similar to the non-AAA group. A closer look to the results of the histological analysis reveals that these differences from the other mice in the AAA group have a basis in the structural tissue composition (cf. Fig. 4). Both cross sections of M2 are characterized by a high content of intact elastin in the wall and large amounts of RBCs within the thrombus, a region which was correlated with strain values comparable to intact elastin without thrombus attachment in the component-wise analysis. Position 1 of M3 does not show any thrombus, with a large section consisting of intact elastin. In Position 2, the lumen is surrounded in part by (1) intact and fragmented elastin without thrombus attachment and (2) thin layers of thrombus with and without RBCs, such that an overall distensible behavior of the wall, reflected by large strain values, was plausible. Note that despite the structural similarity of M2 and M3 to the non-AAA group, the SDIs clearly separate them from the latter. These findings further illustrate that the complex kinematics of aortic dissections is impacted by both hemodynamic forces and tissue composition.

As mentioned above, classical stress-based approaches for the estimation of rupture risk and disease progression are subject to model assumptions as the necessary quantities cannot be determined in vivo. A prerequisite for stress-based approaches is knowledge about the inhomogeneous wall thickness (which is difficult to measure with sufficient accuracy in vivo). Indeed, an accurate description of AAA lumen and wall geometry is one of the most important factors towards the robust prediction of individual material behavior and stress in the vessel wall (Gasser et al. 2022). If the deformed geometry and blood pressure are known, then the stresses can be calculated independently of the material (Joldes et al. 2016). Not knowing inhomogeneous wall thickness in vivo necessitates the assumption of a constant wall thickness, after which the stresses then depend only on the principal radii of curvature (i.e., Laplace’s law where for a given pressure the radius is tightly coupled to a stress value). Biomechanical simulations do offer the advantage that local radii can be used instead of the maximum radius. However, the informative value of these simulations is then almost identical to the maximum diameter criterion currently used. Information about the condition of the vessel wall (degenerated media, proportion of collagen, etc.) cannot be taken into account in this approach and consequently have no influence on the calculated stresses. Although we do not know the condition of the tissue when measuring local deformations, the local deformations themselves are strongly dependent on the condition or degeneration of the wall, since tissue strain under physiological loading is closely related to the tissue’s elastic biomechanical properties. Romo et al. (2014) showed that the site of maximum stress is not equal to the site of maximum strain, but thoracic aortic aneurysms tend to rupture in areas of maximum strain (Romo et al. 2014). Measured strains thus contain much more information than just variation in local geometry. However, what has not been studied in detail is the interpretation of local strains. For example, relatively healthy wall regions with high elastin content and very thin, severely degenerated wall regions may show similarly high strains. Also, as we observed here that the strains of intact elastin and thrombus with RBCs are similar (cf. Fig. 5c A1, A4), although they are two different materials and the latter has almost no mechanical resistance under compressive loading compared to elastin. Therefore, studies such as this one which shed more light on these relationships are needed.

In conclusion, this study revealed the SDIs were capable of distinguishing diseased from non-diseased aortae due to significant structural differences within the AAA group. These results suggest that it will be possible to use 4D ultrasound and strain distribution indices to better monitor changes in the structural composition of of human aneurysms and dissections and obtain additional information on disease progression. Possibly, future work will be able to establish a relation between SDI values and rupture risk. Further studies will be needed to collect a sufficiently large sample of human data to identify such metrics and thresholds. Limitations of this study such as the small sample size and the inherent differences between experimental animal models and human abdominal aortic aneurysms have already been addressed in Cebull et al. (2019). However, our study design that utilized a combination of in vivo imaging, strain mapping, and ex vivo histological quantification necessitated the use of a murine model to establish and optimize this novel multi-modality approach. Moreover, these differences between murine model and human dissecting aneurysms only impair one of the objectives of our current study: A complete understanding of the pathological mechanisms cannot be fully translated from this study to human pathology. The second methodological goal of the study was to determine how 4D ultrasound strain imaging can be used to identify and distinguish tissue components with characteristically different elastic properties within the aneurysm wall and thrombus noninvasively in vivo. This goal is not at all affected by the differences between the animal model and human AAA since the identical relevant tissue components are found in both: intact and fragmented elastin, young thrombus that is rich of erythrocytes and fibrin, and old thrombus. In other studies, the in-plane strains in the wall were usually calculated to distinguish or characterize local variations of wall tissue (Aggarwal et al. 2022; Bersi et al. 2016, 2019; Derwich et al. 2016; Forneris et al. 2020; Satriano et al. 2015; Wittek et al. 2018).

To our knowledge, the current study is the first to quantitatively assign not only different regions of the wall, but different tissue types such as intact and fragmented elastin using in vivo strain imaging. These findings build upon our previous work where the results showed that presence of IMT and focal breakage in the medial elastin significantly reduced mean aortic strain (Cebull et al. 2019). The analysis from this current study was able to differentiate types of thrombus and vessel wall components. Taken together, our findings suggest that the application of 4D ultrasound imaging and strain mapping to human patients could provide valuable information for a better understanding and evaluation of the disease progress.

### Supplementary Information

Below is the link to the electronic supplementary material.

Supplementary file 1 (pdf 127898 KB)

## Figures and Tables

**Fig. 7:**
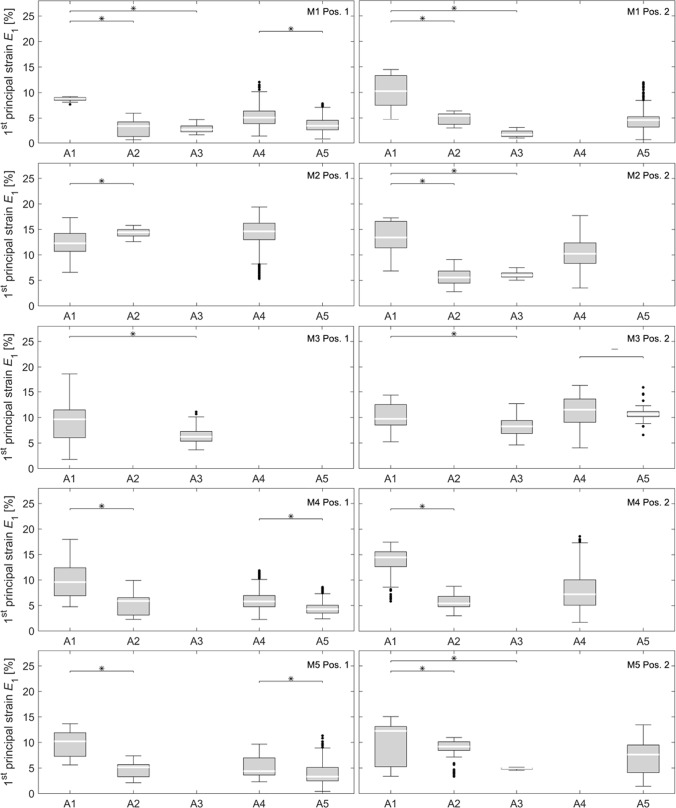
Box-Whisker plots of strains in the differentiated aneurysm areas (A1–A5) of all single section positions (Pos. 1 and Pos. 2) of all five mice (M1–M5). A1 and A2 describes the regions in elastin without and with adjacent thrombus (“Elastin w/o” and “w/ thrombus”). A3 describes the regions where fragmented elastin is present “Fragmented (Frag.) Elastin”. Similarly, A4 and A5 describes regions with varying levels of erythrocytes within the intramural thrombus “thrombus w/” and “w/o red blood cells (RBCs)”. * indicates a significant difference between groups at the 5% level with Bonferroni correction, - indicates no significant difference

## Appendix A: Strain distributions from individual sections

Box-Whisker plots of strains in the differentiated aneurysm areas (A1–A5) for all ten single sections (Pos. 1 and Pos. 2) of the five mice (M1-M5) studied are shown in Fig. 7. Figure 5 shows the same strain distributions when strains of these ten individual sections are combined.

## Appendix B Strain distributions of the error estimation

The resulting strains in the differentiated aneurysm regions based on the FE analyses with constant and varied material parameter μ are shown in Fig. 8.

## Declarations

### Availability of data and materials

Data available upon request.
