# Supplementary material for: Biomechanical characterization of tissue types in murine dissecting aneurysms based on histology and 4D ultrasound-derived strain
Source: Biomech Model Mechanobiol. 2023 Sep 14;22(5):1773–88. doi: 10.1007/s10237-023-01759-6 (PMC10511389; doi:10.1007/s10237-023-01759-6)
Supplement: Supplementary file 1 — (pdf 127898 KB) [file 10237_2023_1759_MOESM1_ESM.pdf]

Supplemental Data

of

Biomechanical Characterization of Tissue Types in Murine Dissecting Aneurysms based on Histology and 4D Ultrasound-Derived Strain

by

Achim Hegner, Hannah L. Cebull, Antonio J. Gamez, Christopher Blase, Craig J. Goergen and Andreas Wittek

1 Histological images

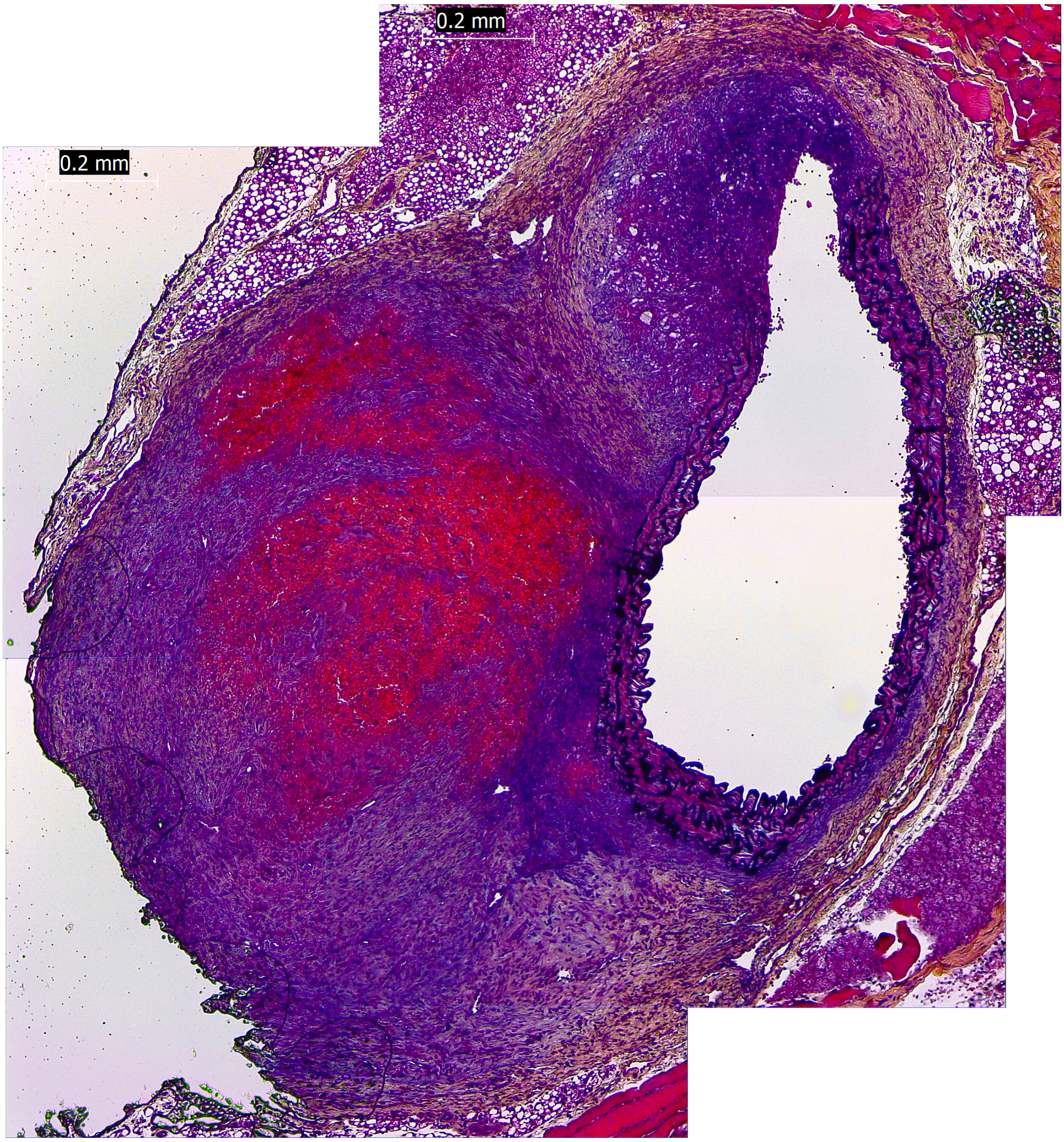

Figure 1.1: M1 Pos3 Histo 10x Movat

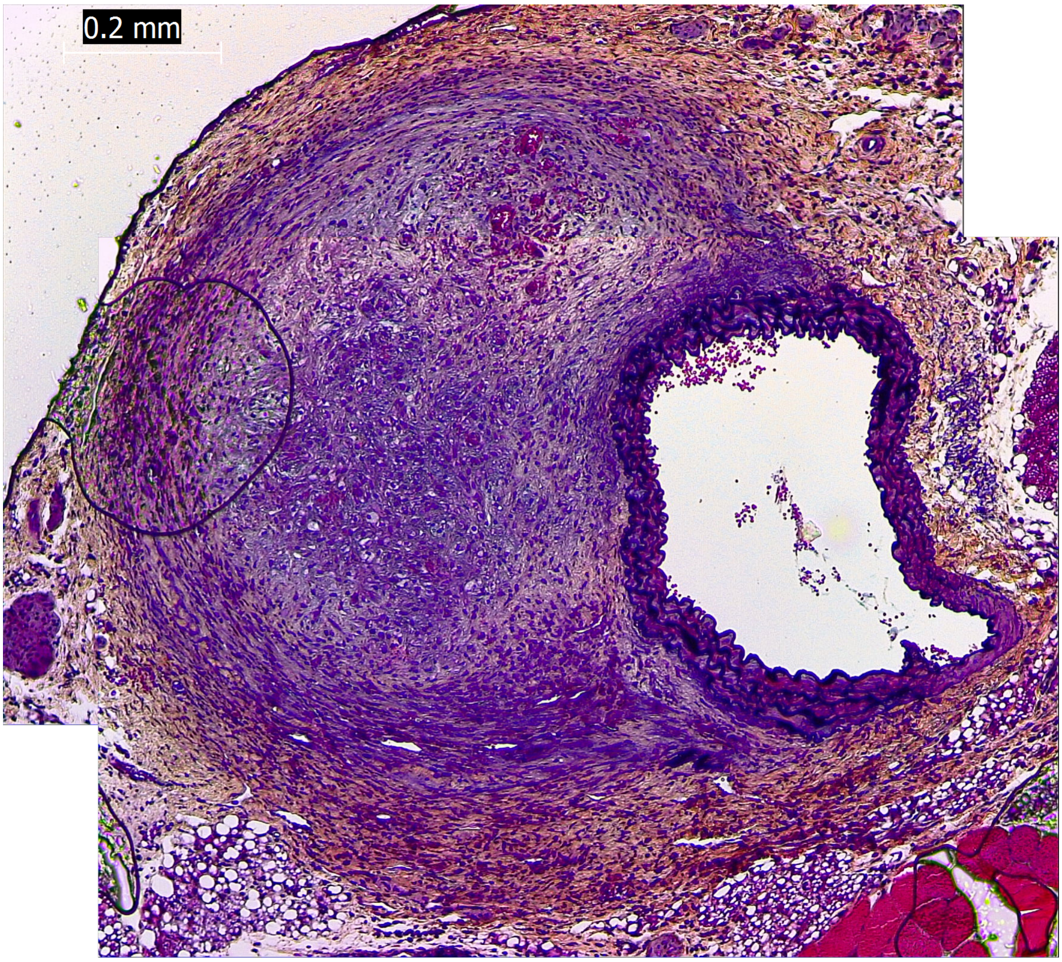

Figure 1.2: M1 Pos4 Histo 10x Movat

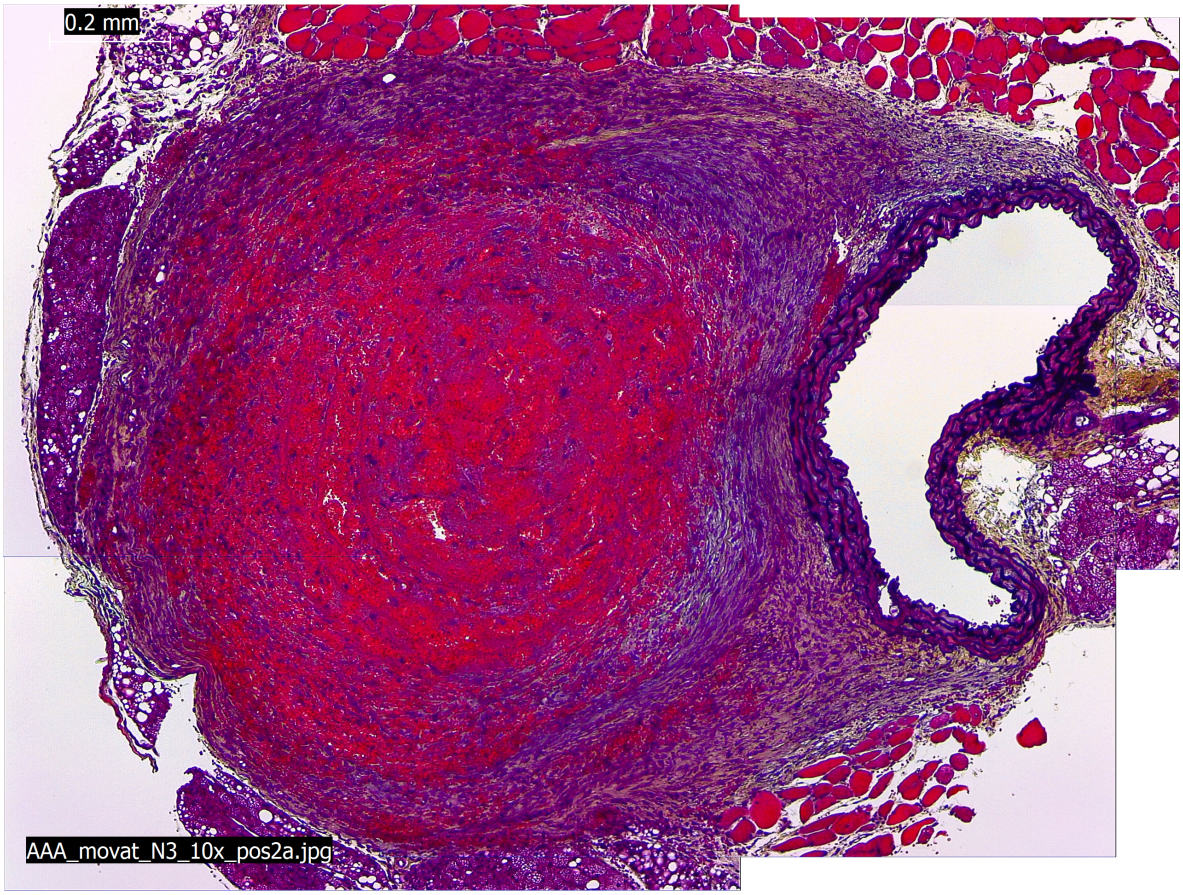

Figure 1.3: M2 Pos2 Histo 10x Movat

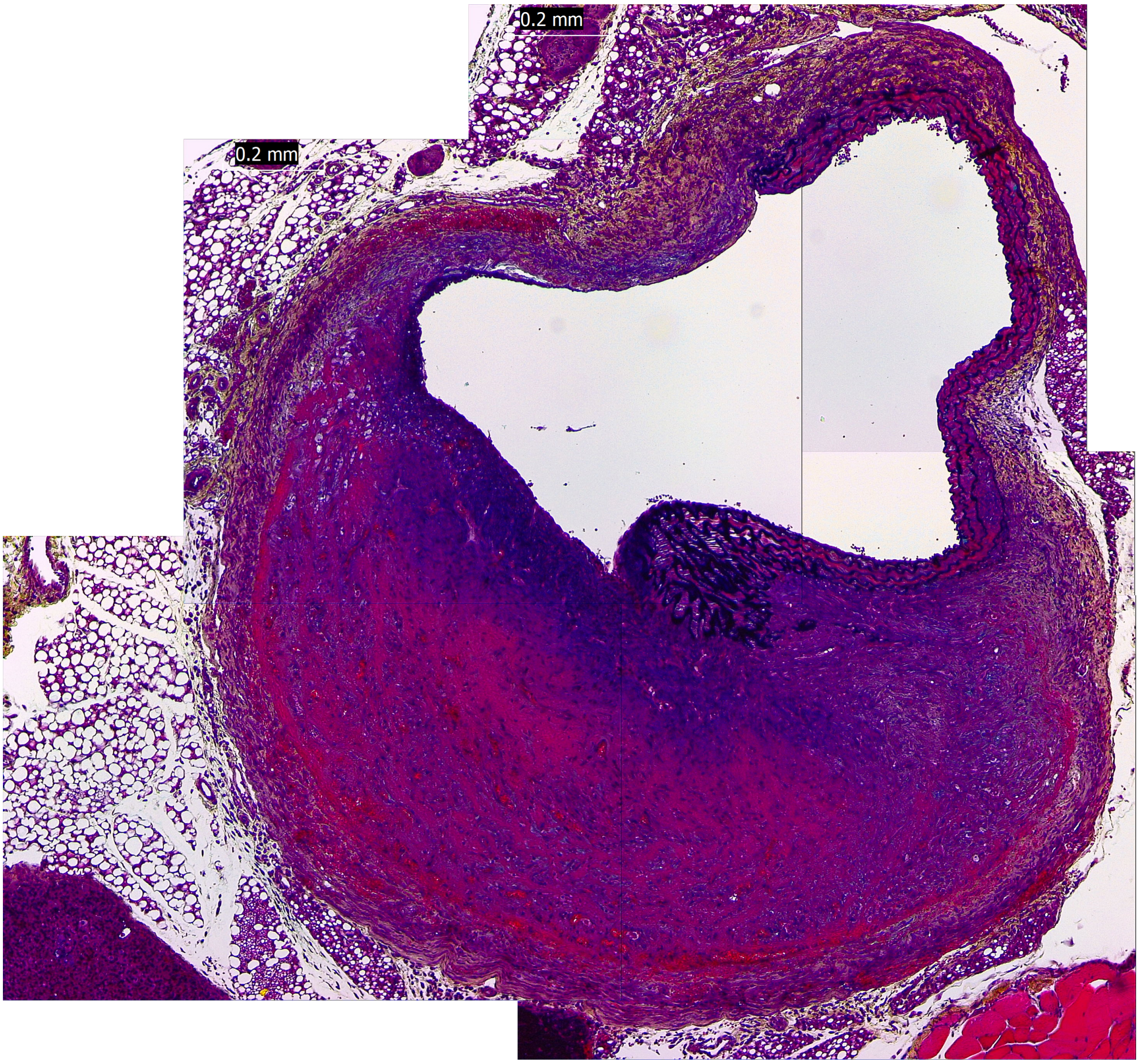

Figure 1.4: M2 Pos3 Histo 10x Movat

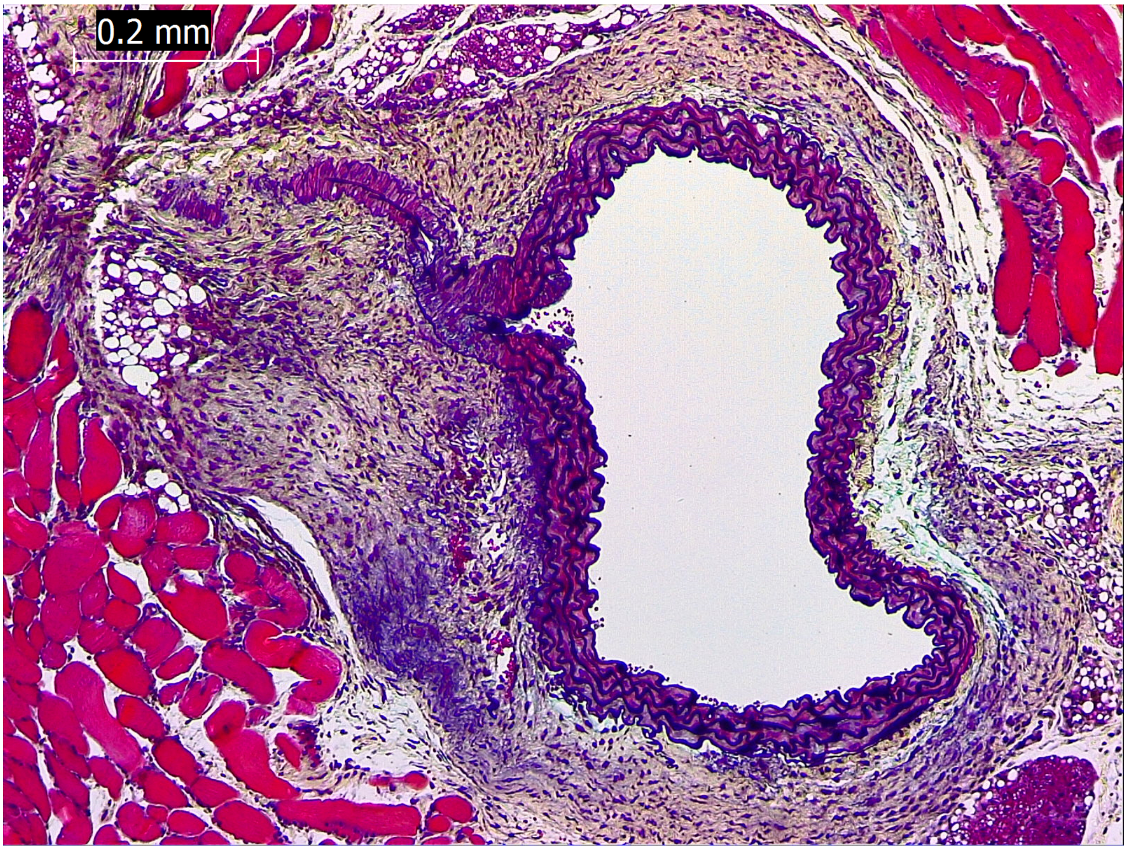

Figure 1.5: M3 Pos2 Histo 10x Movat

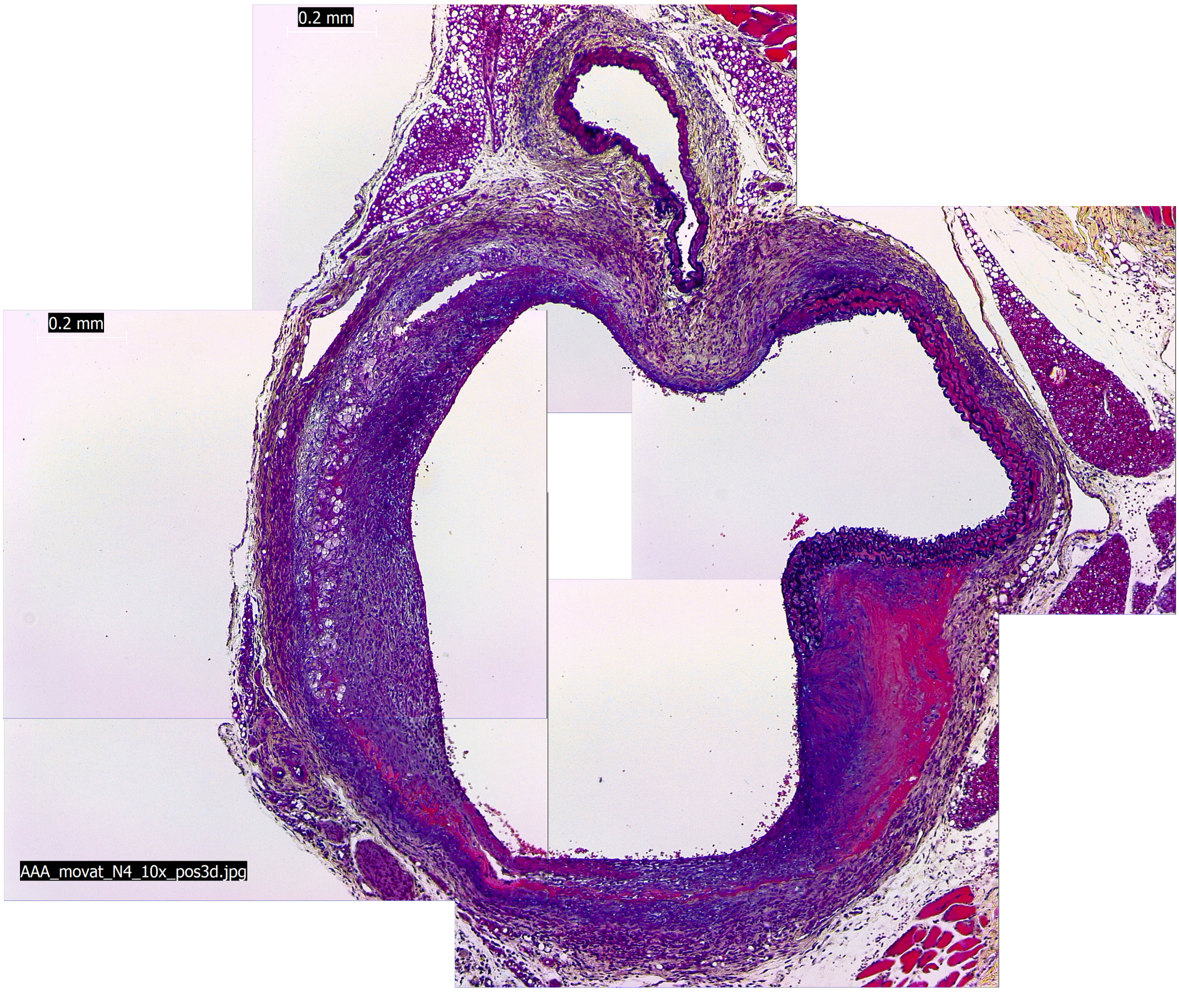

Figure 1.6: M3 Pos3 Histo 10x Movat

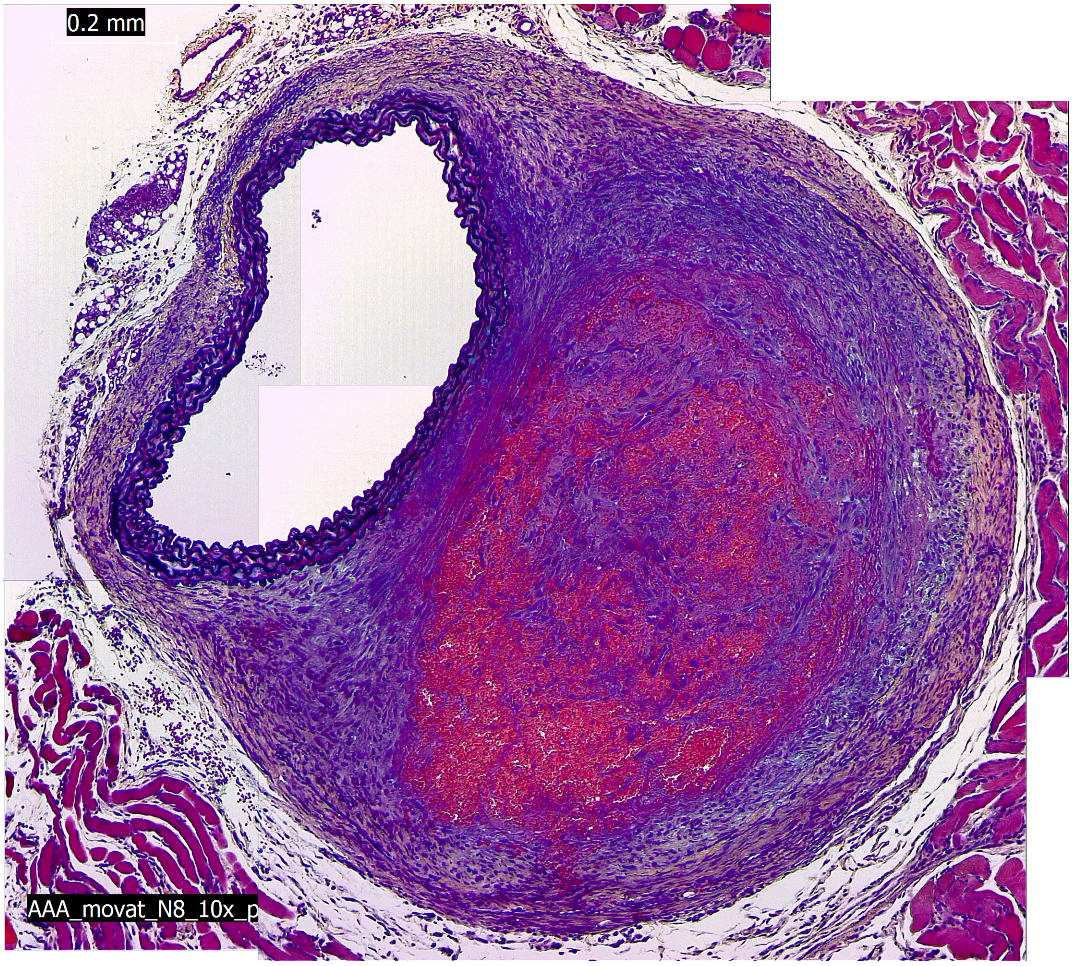

Figure 1.7: M4 Pos1 Histo 10x Movat

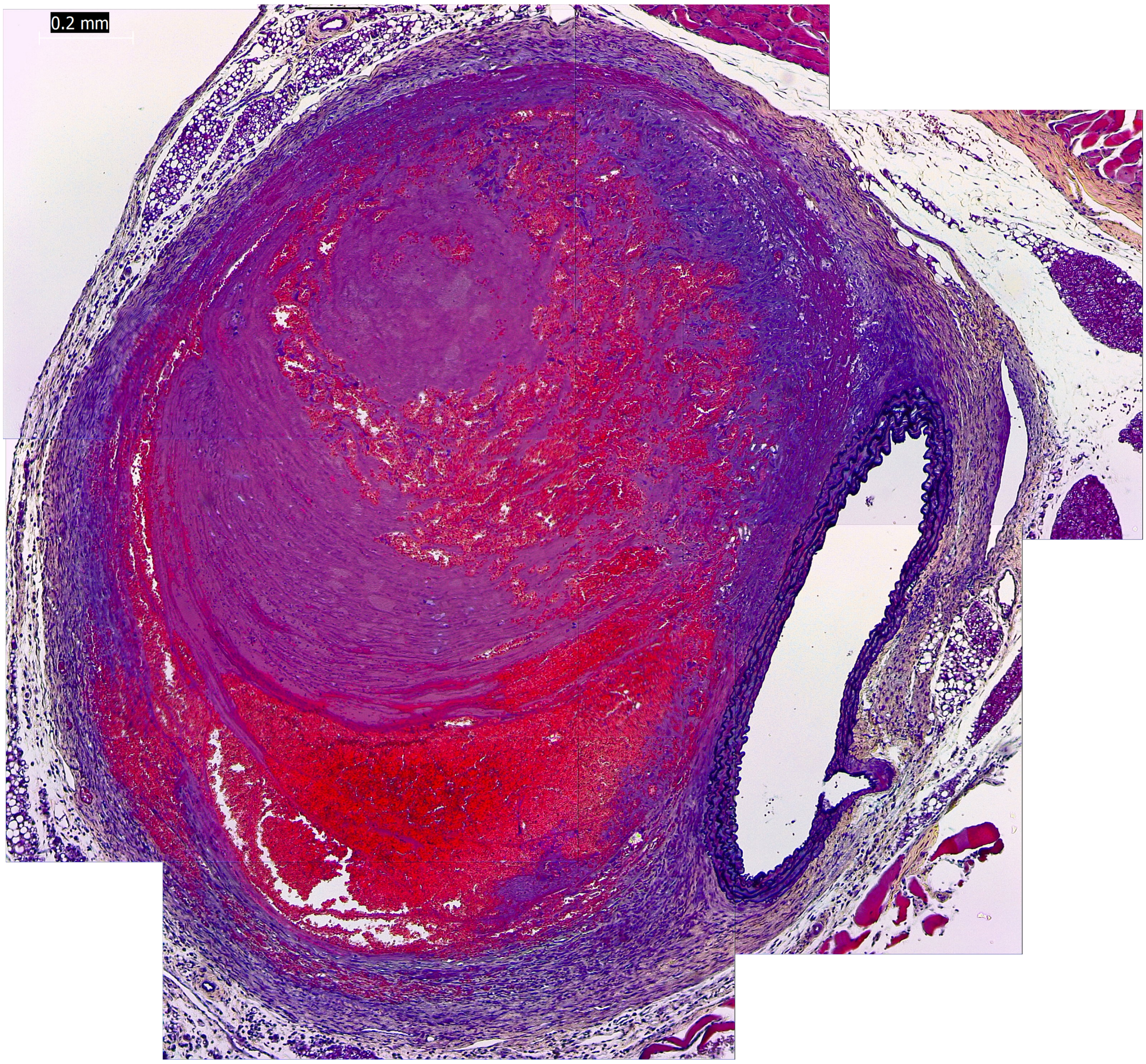

Figure 1.8: M4 Pos2 Histo 10x Movat

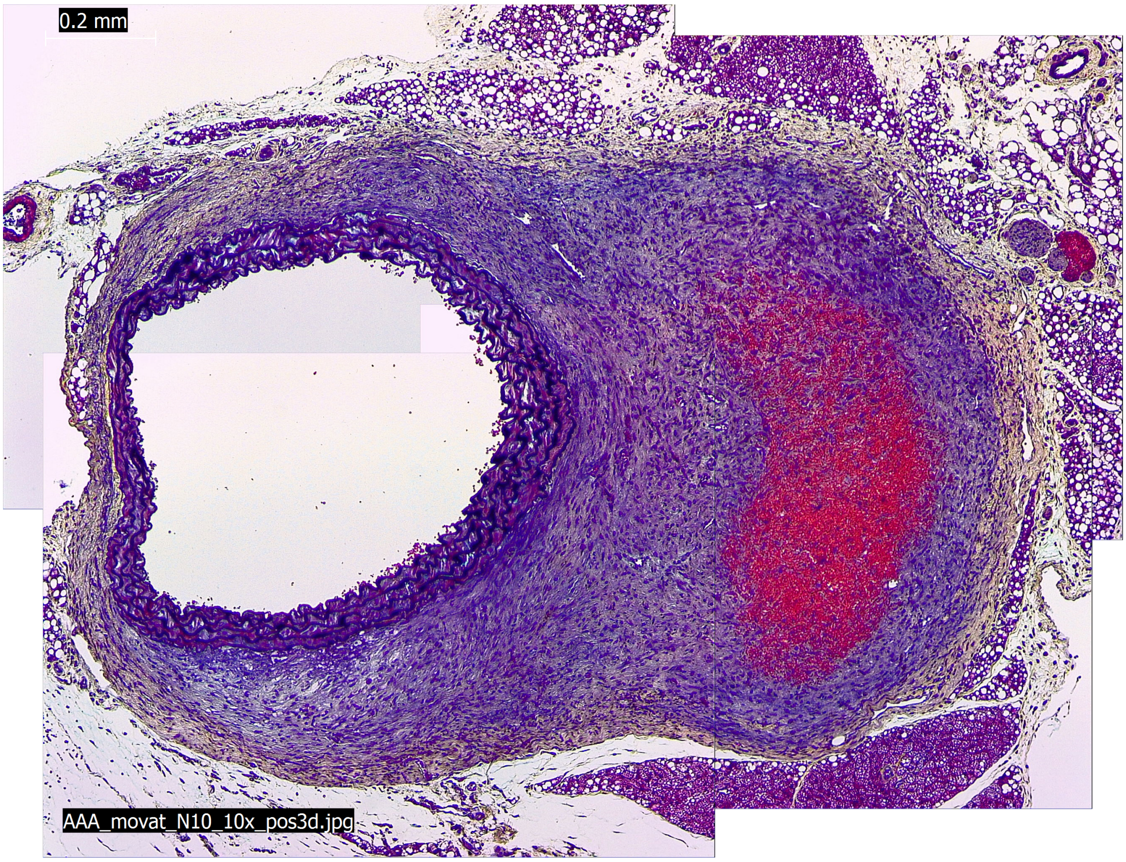

Figure 1.9: M5 Pos3 Histo 10x Movat

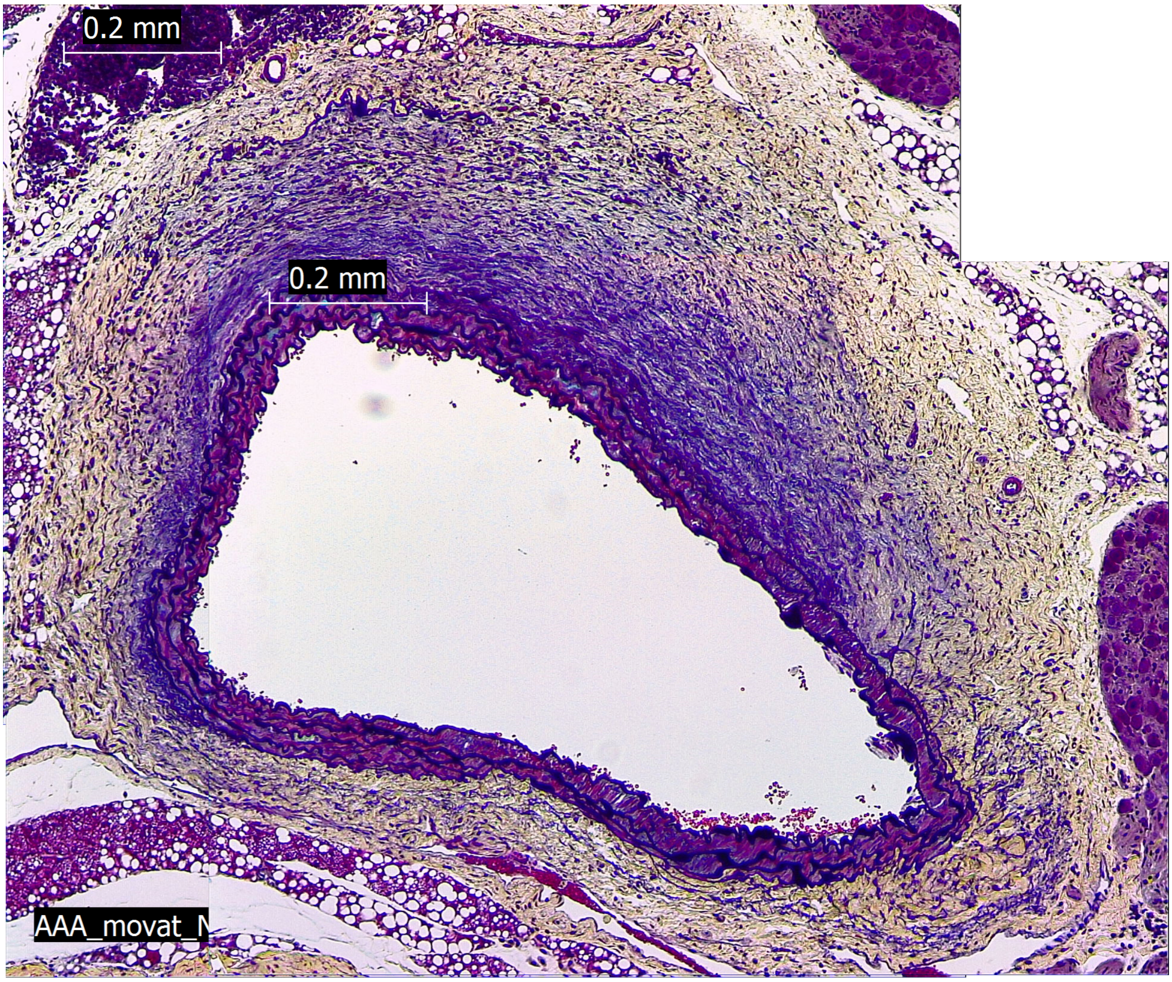

Figure 1.10: M5 Pos4 Histo 10x Movat
